# A (-)-Carvona Modula a Sinalização de Cálcio Intracelular com Ação Antiarrítmica em Corações de Ratos

**DOI:** 10.36660/abc.20210499

**Published:** 2022-07-29

**Authors:** Gilmara Beatriz Andrade da Silva, Diego Santos Souza, José Evaldo Rodrigues Menezes-Filho, Júlio Alves da Silva-Neto, Jader dos Santos Cruz, Danilo R. Roman-Campos, Lucindo José Quintans-Júnior, Carla Maria Lins de Vasconcelos

**Affiliations:** 1 Universidade Federal de Sergipe São Cristóvão SE Brasil Universidade Federal de Sergipe, São Cristóvão, SE – Brasil; 2 Universidade Federal de Minas Gerais Instituto de Ciências Biológicas Belo Horizonte MG Brasil Universidade Federal de Minas Gerais – Instituto de Ciências Biológicas, Belo Horizonte, MG – Brasil; 3 Universidade Federal de São Paulo São Paulo SP Brasil Universidade Federal de São Paulo, São Paulo, SP – Brasil

**Keywords:** Arritmias Cardíacas, Monoterpenos, Ratos

## Abstract

**Fundamento:**

A (-)-carvona é um monoterpeno encontrado em óleos essenciais com atividade antioxidante e anti-inflamátoria.

**Objetivos:**

O objetivo deste estudo foi analisar a propriedade antiarrítmica da (-)-carvona no coração de rato e seus efeitos sobre a sinalização de Ca^+2^ intracelular.

**Métodos:**

Os efeitos da (-)-carvona foram avaliados sobre a contratilidade atrial (0,01 – 4 mM) e ventricular (0,5 mM), e no eletrocardiograma (0,5mM). A fração de encurtamento, a corrente de cálcio do tipo L (I_Ca,L_) e a sinalização de Ca^+2^ foram medidas no cardiomiócito isolado (0,5 mM). O efeito antiarrítmico foi avaliado no modelo de arritmia induzida por sobrecarga de cálcio (0,5 mM) (n = 5). Um p < 0,05 foi adotado como nível de significância estatística.

**Resultados:**

No átrio, a (-)-carvona causou inotropismo negativo de maneira concentração-dependente (EC_50_ 0,44 ± 0,11 mM) e diminuiu o inotropismo positivo induzido pelo CaCl_2_ (0,1 – 8,0 mM) e BAY K8644 (5 - 500 nM), um agonista de canal de cálcio do tipo L. Em coração isolado, a (-)-carvona (0,5mM) reduziu a contratilidade ventricular em 73% e a frequência cardíaca (em 46%), aumentou o Pri (30,7%, tempo desde o início da onda P até a onda R) e o QTc (9,2%, uma medida de despolarização e repolarização dos ventrículos), sem mudar a duração do complexo QRS. A (-)-carvona diminuiu a fração de encurtamento (61%), a (I_Ca,L_) (79%) e o transiente intracelular de Ca^+2^ (38%). Além disso, a (-)-carvona apresentou ação antiarrítmica, identificada pela redução do escore de arritmia (85%) e ocorrência de fibrilação ventricular.

**Conclusão:**

A (-)-carvona reduz a entrada de Ca^+2^ através de canais de Ca^+2^ do tipo L e, assim, diminui a contratilidade cardíaca e o Ca^+2^ intracelular e apresenta promissora atividade antiarrítmica no coração de ratos.

## Introdução

As arritmias são consideradas um problema sério de saúde pública e são uma importante causa de morbidade e mortalidade no mundo.^1^ Entre as principais, batimentos ventriculares prematuros (BVP), taquicardia ventricular sustentada, e fibrilação ventricular são comuns em pacientes com miocardiopatia isquêmica ou não isquêmica.^1^ No entanto, os tratamentos com drogas antiarrítmicas geralmente causam respostas pró-arrítmicas adversas ou ausência de melhora na qualidade de vida de indivíduos com arritmias.^2^

Desde 1970, quando Vaughan-Williams classificou as drogas antiarrítmicas com base em seus mecanismos farmacológicos para bloquear canais iônicos ou receptores específicos, pesquisadores investiram muito tempo e esforço para descobrir novas terapias com um risco mais baixo de efeitos adversos para o paciente.^3–5^

Entre as novas terapias, compostos de origem natural têm demonstrado sua capacidade de inibir arritmias cardíacas ventriculares, gerando interesse na comunidade científica. Os terpenos mostraram-se como os principais compostos com atividade antiarrítmica comprovada.^6–9^ Entre eles, a carvona (*p*-mentha-6,8-dien-2-one) é de particular interesse, dadas suas propriedades já estabelecidas. A carvona é uma cetona pertencente ao grupo dos monoterpenos, e conhecida por sua atividade antioxidante, antimicrobiana, e antifúngica.^10,11^ Parece atuar também sobre canais de sódio dependentes de voltagem nos neurônios, levando a um efeito anticonvulsionante.^12,13^ Ainda, a carvona demonstrou um efeito antiespasmódico pela inibição dos canais de cálcio dependentes de voltagem, e uma ação anticâncer sinergística com a doxorrubicina sobre células MCF7, reduzindo sua cardiotoxicidade.^14^ Como já foi decrito na literatura, a carvona bloqueia canais de sódio e cálcio, e medicamentos dessa classe têm propriedades antiarrítmicas e cardioprotetoras. Assim, decidimos estudar os efeitos da carvona sobre a regulação do cálcio na célula e sua possível ação antiarrítmica nos corações de ratos.^9^

Embora existam vários estudos sobre a (-)-carvona na literatura científica, em nosso conhecimento, não existem explicações ou hipóteses sobre o mecanismo de ação desse monoterpeno no músculo cardíaco. Portanto, nosso objetivo foi avaliar os possíveis efeitos cardíacos da (-)-carvona, e apresentar uma melhor explicação científica de suas ações no tecido cardíaco que possa servir como base para o desenvolvimento de novos medicamentos de origem natural para o tratamento de arritmias.

## Materiais e métodos

### Animais

Os experimentos foram conduzidos com ratos machos Wistar (250-300 g) obtidos do biotério da Universidade Federal de Sergipe (UFS). Em cada procedimento, foram usados cinco animais.^15^ Este estudo foi aprovado pelo Comitê de ética em pesquisa com animais da UFS (protocolo 61/16, 20 de fevereiro de 2017). O manejo dos ratos foi realizado em acordo com os princípios éticos na experimentação animal (NIH publication 86-23, revised 1985; http://oacu.od.nih.gov/regs/index.htm).

### Avaliação do efeito inotrópico da (-)-carvona

O efeito inotrópico da (-)-carvona foi avaliado no átrio esquerdo dos corações dos ratos imersos em uma câmara contendo solução de Krebs-Henseleit (K-H) (em mM): NaCl 120, KCl 5,4, MgCl_2_ 1,2, NaHCO_3_ 27, CaCl_2_ 1,25, Glicose 10, NaH_2_PO_4_ 2,0 (pH 7,4). O átrio foi mantido a 29° ± 0,1°C, oxigenado (95% O_2_ e 5% CO_2_), estirado a uma tensão de 5mM e submetido à estimulação de campo (1 Hz, 100 V, 0,5 ms) (Stimulator SD9 GRASS). A força atrial foi registrada utilizando um transdutor de força isométrica (GRASS FT03), e os sinais foram digitalizados (DATAQ DI710, WINDAQ PRO Acquisition). As curvas de dose-resposta da (-)-carvona (0,001 – 4,0 mM) e nifedipina (0,03 - 100 µM, bloqueador de canal de Ca^+2^) foram obtidas para determinar a resposta contrátil e calcular o EC50. Dimetilsulfóxido (DMSO) 0,5% foi usado como diluente para a (-)-carvona.

#### Efeitos da (-)-carvona sobre a entrada de cálcio no miocárdio atrial

Para analisar a ação da (-)-carvona sobre a entrada de Ca^+2^, foram obtidas curvas de dose-resposta de CaCl_2_ (0,1 a 8,0 mM) e (±)-Bay K8644 (5 a 500 nM) no átrio esquerdo no controle e após pré-incubação com (-)-carvona (1 mM) por 15 minutos. Os resultados foram expressos em porcentagens da resposta contrátil máxima do átrio ao CaCl_2_ no controle. Em ambos os protocolos, a concentração inicial de CaCl_2_ na solução de K-H foi 0,5mM.^7,16^

#### Effeitos da (-)-carvona sobre o perfil eletrocardiográfico e pressão do ventrículo esquerdo (PVE)

Após a administração intraperitoneal de heparina (1000 UI) por 15 minutos, os corações foram removidos e preparados em um sistema de perfusão aórtica de fluxo constante (10mL/min). O coração foi perfundido com solução de K-H previamente filtrada (0,45 µm), oxigenado (95% O_2_ + 5% CO_2_) e mantido a 34 ± 0,1°C (Haake F3). Para registrar o eletrocardiograma (ECG), três eletrodos (Ag/AgCl/NaCl 1 M) foram colocados sobre o coração para detectar sinais elétricos. Os sinais foram amplificados e digitalizados (PowerLab 4/35 ADInstrument, EUA). A medida da PVE foi realizada utilizando-se um balão com água (15cm/Hg) introduzido na cavidade do ventrículo esquerdo. Esse aparelho foi acoplado a um transdutor de pressão (MLT0699/A). Os sinais foram amplificados e enviados para um conversor AD (PowerLab 4/35 26 ADInstrument, EUA). O sistema foi calibrado usando uma coluna de mercúrio. Os parâmetros de contração (PVE, tempo para o pico e tempo de relaxamento) foram avaliados em 30 batimentos consecutivos pelo programa LabChart 8.0 Pro Software (ADInstruments, USA) em situação controle e após cinco, 10 e 15 minutos após o início da perfusão com carvona (0,5 mM). O ECG mediu o intervalo PR (PRi – período entre o início da onda P até a onda R), duração do complexo QRS (QRS – período que se estende da onda Q até a onda S), e o intervalo QT (QTi - período entre o início da onda Q até a onda o final da onda T). O QTi foi convertido a QTc usando a formula de Bazett normalizada para roedores (QTc-B = QTi/*RR*/*f*), onde f é a duração média do intervalo RR no controle (f = 271 ms).

#### Efeitos da (-)-carvona sobre a fração de encurtamento

Os cardiomiócitos do ventrículo esquerdo e direito foram isolados dos ratos de acordo com o protocolo de Shioya (2007),^17^ com algumas modificações. A fração de encurtamento foi avaliada medindo-se a mudança no comprimento celular com um microscópio invertido acoplado a um sistema de detecção de bordas (Ionoptix, EUA). Os cardiomiócitos foram colocados em uma câmera experimental (temperatura ambiente) contendo solução de Tyrode (em mM: NaCl 150, KCl 5,4, MgCl_2_ 0,5, HEPES 10, Glicose 10, CaCl_2_ 1,8, pH 7,4). Os cardiomiócitos foram visualizados usando uma câmera (Ionoptix Myocam at 240 Hz) acoplada a um microscópio e um programa de detecção de imagem foi utilizado (Ionoptix Ionwizard 6,3). As mudanças longitudinais nas bordas dos cardiomiócitos foram capturadas pelo sistema de detecção, e os dados gerados foram armazenados e analisados. A fração de encurtamento foi avaliada nas células controles e após incubação com (-)-carvona 0,5mM.

#### Efeitos da (-)-carvona sobre a corrente de cálcio do tipo L (I_Ca,L_)

Os registros de ‘voltage-clamp’ do grampeamento de voltagem foram obtidos usando um EPC 10.2 (HEK Elektronik, Alemanha). Na configuração de célula inteira, aguardou-se um período de 3-5 minutos para se estabelecer o equilíbrio iônico entre a pipeta e o meio intracelular. Os eletrodos de registro possuíam resistência de 2-3 MΩ. Os cardiomiócitos ventriculares com resistência de série acima de 8 MΩ foram descartados. A composição da solução interna era (em mM): 120 CsCl, 20 TEACl, 5 NaCl, 10 HEPES e 10 EGTA, 1 MgCl2 (pH foi estabelecido em 7,2 usando CsOH), e a solução externa foi composta de (em mM): 150 TEACl, 0,5 MgCl2, 1,8 CaCl2, 10 HEPES e 11 glicose (pH 7,4, com TEAOH). Para avaliar os efeitos agudos de 0,3 e 0,5 mM de (-)-carvona sobre I_Ca,L_, registrou-se um curso temporal do pico da corrente I_Ca,L_ tanto na ausência como após exposição a uma data concentração de (-)-carvona. Foram aplicados pulsos com potenciais entre −80 mV to −40 mV por 50 ms para inativar quaisquer canais de Na^+^ ou do tipo Ca^+2^ remanescentes. Em seguida, foi aplicado um pulso de teste a 0mV por 300ms para medir I_Ca,L_.

#### Efeitos da (-)-carvona sobre o transiente global de Ca^+2^ intracelular

Os cardiomiócitos do ventrículo direito e esquerdo foram incubados com 10 µM de FLUO4-AM (Molecular Probes, Eugene, OR, EUA) diluídos com DMSO por 30 minutos. As células em seguida foram lavadas com solução de Tyrode (1,8 mM Ca^+2^) para retirar o excesso de FLUO4-AM. Um sistema confocal (Zeiss GmbH, Jena, Alemanha) com uma objetiva de imersão em óleo (63x) foi usado para análise de imagens fluorescentes confocais. O FLUO4-AM foi excitado a 488nm (laser de argônio) e a intensidade da emissão foi medida a 510 nm. Os cardiomiócitos foram escaneados com uma linha de 512 pixel posicionada ao longo do eixo longitudinal da célula, a cada 1,54 ms. O processamento da imagem digital foi realizada usando uma linguagem de programação IDL (Research Systems, Boulder, CO, EUA).^9^ Os níveis de Ca^2+^ intracellular foram expressos em F/F_0_, onde F_0_ indica a fluorescência de repouso. O transiente global de Ca^+2^ intracelular foi registrado no controle e após três minutos de incubação com (-)-carvona 0,5mM à temperatura ambiente.

### Efeitos antiarrítmicos da (-)-carvona

Arritmia *ex-vivo* foi determinada em corações isolados como descrito previamente.^18^ Primeiramente, os corações foram perfundidos com solução K-H contendo 1,25 mM de cálcio (grupo controle). Após 20 minutos, os corações foram perfundidos com solução K-H contendo 3,3 mM de cálcio (grupo com cálcio elevado) ou com cálcio +0,5 mM (-)-carvona durante 15 minutos (grupo cálcio elevado + carvona). O ECG foi monitorado por 15 minutos para avaliar a ocorrência de arritmias. As arritmias encontradas foram BVP, taquicardia ventricular (TV) e fibrilação ventricular (FV). O período de 15 minutos de experimento foi dividido em intervalos de três minutos e os escores de arritmia foram adicionados no final, como descrito por Curtis e Walker (1988).^9,19^ Episódios de BVP < 3 eventos/3 minutos foram classificados como escore 0 e > 10 eventos/3 minutos receberam escore 1; 1-5 episódios de TV < 40 s receberam escore 2, e > 5 episódios de TV ou um episódio de FV com duração < 40 s receberam escore 3; 2 - 5 episódios de TV ou FV com duração < 80 s receberam escore 4; > 5 episódios de FV, TV e/ou FV com duração < 160 s receberam escore de 5; TV e/ou FV com duração < 300 s receberam escore de 6, e duração > 300 s escore 5.

### Análise estatística

Todos os resultados foram apresentados em média ± desvio padrão (DP). O programa GraphPad Prism v.5.0 (GraphPad Software, CA, USA) foi usado para as análises. A normalidade dos dado foi testada usando o teste de Shapiro-Wilk. Os valores médios foram comparados usando a análise de variância (ANOVA) seguida do teste post hoc de Tukey ou teste t não pareado. Um p<0,05 foi usado como nível de significância.

## Resultados

**Figura 1:**
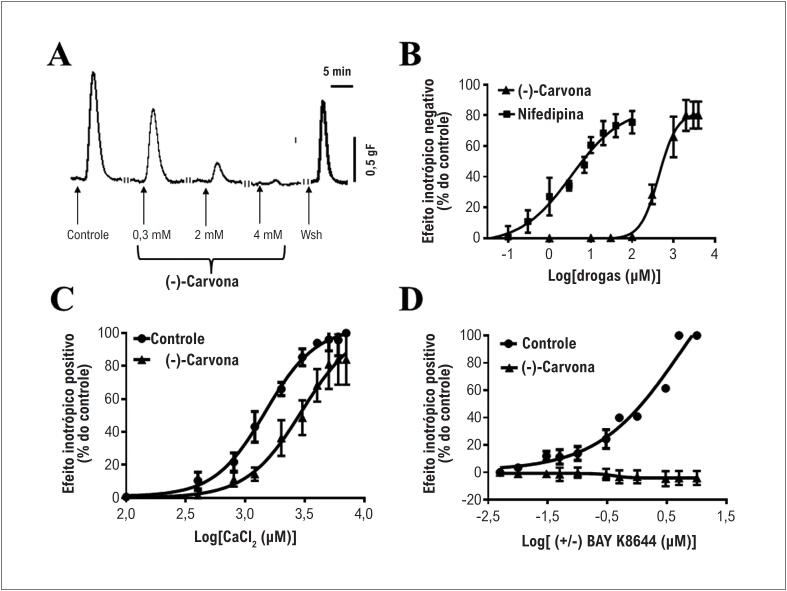
A (-)-carvona exibiu efeito inotrópico negativo e diminuiu a entrada de cálcio no átrio esquerdo de rato. (A) Traçados experimentais da contração atrial isolada no controle, após incubação com (-)carvona (0,3, 2 e 4mM) e *washout* (Wsh); (B) Curvas de concentração-resposta do inotropismo negativo da (-)carvona e nifedipina (bloqueador de canal de cálcio); (C) e (D) curvas de concentração-resposta do CaCl_2_ e (±)-BAY K8644 na ausência e presença de 1 mM de (-)-carvona, respectivamente (n = 5).

A (-)-carvona (0,003 a 4 mM) reduziu a força atrial de maneira concentração-dependente. A Figura 1A mostra traçados de curvas da concentração atrial isolada em situação controle, com (-) carvona 0,3, 2 e 4 mM, e no *washout*. Como pode ser visto, 4mM de (-)-carvona reduziram a contratilidade do miocárdio em aproximadamente 96%, e a reversibilidade após o *washout* foi de aproximadamente 65%. A Figura 1B mostra uma curva de concentração-resposta do efeito inotrópico negativo da (-)-carvona que apresentou um EC_50_ de 0,44 ± 0,11 mM (n = 5). A nifedipina, usada como controle positivo, apresentou valores de EC_50_ de 0,0034 ± 0,0011 mM (n = 5). O DMSO a 0,5%, usado como diluente, não apresentou efeito sobre a força atrial (dados não apresentados).

Uma vez que a (-)-carvona provocou um efeito inotrópico negativo, decidimos investigar se o canal de cálcio está envolvido em seu mecanismo de ação. Os resultados revelaram que a (-)carvona (1mM) deslocou a curva de concentração-resposta para a direita, aumentando o EC50 do CaCl_2_ de 1,46 ± 0,14 mM (controle) para 3,17 ± 0,22 mM (CaCl2 + carvona) (Figura 1C, n = 5, p < 0,05). Um dado interessante foi a inibição do inotropismo induzido pelo (±)-BAY K8644, um agonista do canal de cálcio do tipo L (Figura 1 D).

**Figura 2:**
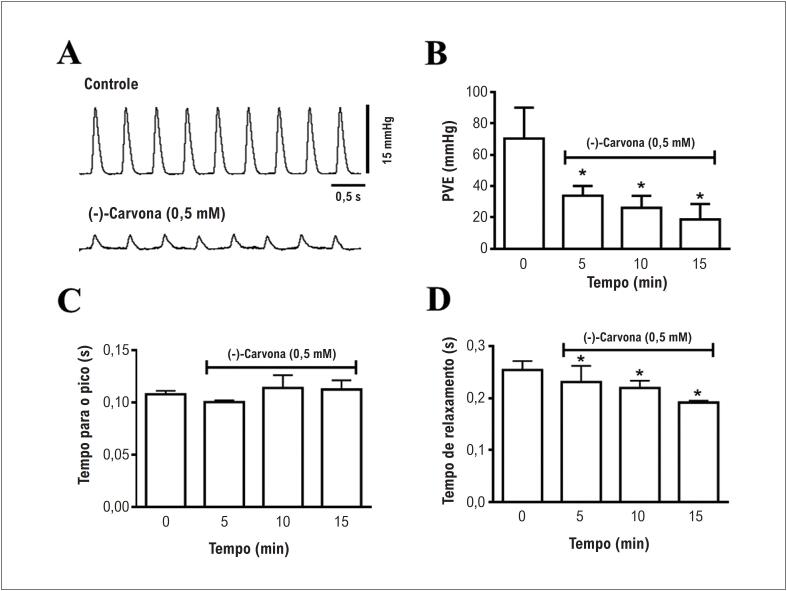
Efeitos da (-)-carvona sobre a contratilidade no coração de rato isolado. (A) registros da pressão do ventrículo esquerdo (PVE) no controle (painel superior) e com 0,5 mM de (-)carvona (painel inferior); (B) PVE; (C) tempo para o pico; e (D) tempo de relaxamento (n = 5, *p < 0,05).

Nos corações isolados, 0,5mM de (-)-carvona também induziu uma redução na PVE, como pode ser observado nos traçados da Figura 2A (n=5). Uma redução de 73% na PVE foi observada após 15 minutos de perfusão do coração com 0,5mM de (-)-carvona (Figura 2B). A (-)-carvona não alterou o tempo para o pico (Figura 2C), mas reduziu significativamente o tempo de relaxamento (24%) após 15 minutos de perfusão com (-)-carvona (Figura 2D).

**Figura 3:**
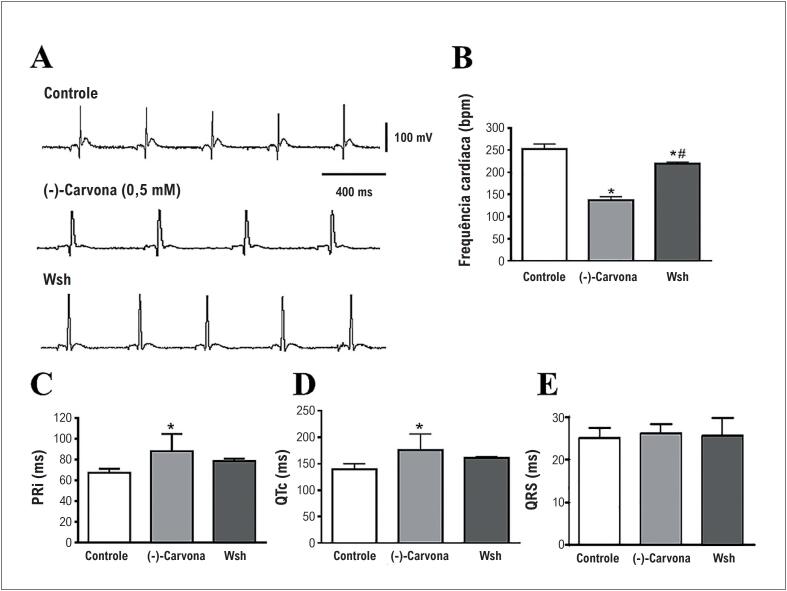
Efeitos da carvona sobre o pefil eletrocardiográfico no coração de rato isolado. (A) Registros eletrocardiográficos no controle, com 0,5 mM de (-)carvona como *washout* (Wsh), (B) Frequência cardíaca; (C) intervalo PR (Pri), (D) intervalo QTc e (E) duração do complexo QRS (n = 5, *p < 0,05 vs controle e #p < 0,05 vs (-)-carvona).

A Figura 3A apresenta traçados ecocardiográficos representativos da situação controle após 15 minutos de perfusão com 0,5mM de (-)-carvona e *washout*. Como pode ser observado, a (-)-carvona diminuiu a frequência cardíaca (n=5, Figura 3B) e aumentou tanto o PRi como o QTi (n=5, Figura 3C e D), sem mudar a duração do complexo QRS (Figura 3E).

**Figura 4:**
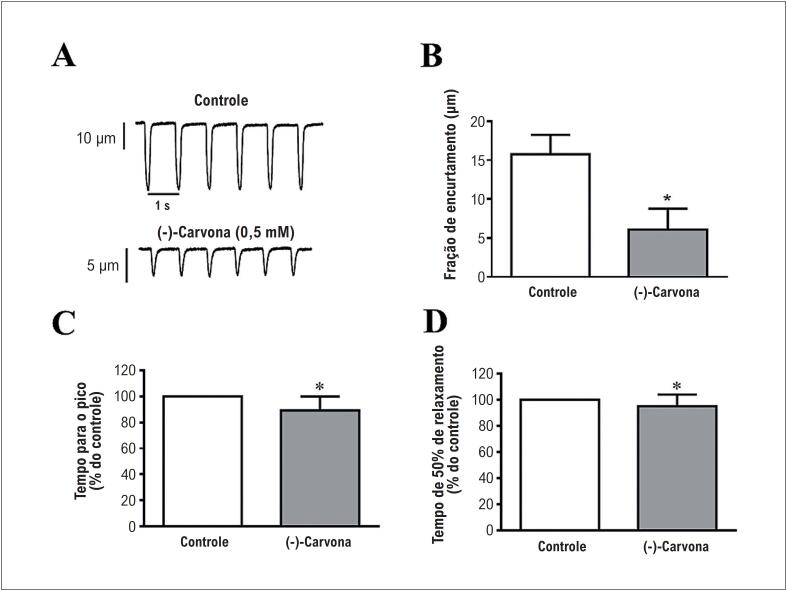
Efeitos da (-)-carvona sobre a fração de encurtamento no cardiomiócitos ventricular isolado. (A) registro da fração de encurtamento no controle (painel superior) e após incubação com 0,5mM de (-) carvona (painel inferior); (B) fração de encurtamento no controle e (-)-carvona; (C) tempo para o pico; (D) tempo para relaxamento de 50% (n=5, *p<0,05).

A Figura 4A apresenta registros da contratilidade celular na situação controle (painel superior) e após perfusão com 0,5mM de (-)-carvona (painel inferior), mostrando a redução na fração de encurtamento nos cardiomiócitos. As médias dos resultados mostraram redução da fração de encurtamento após incubação com (-)-carvona (n=5, Figura 4B). Ainda, a (-)-carvona reduziu tanto o tempo para o pico como o tempo de relaxamento em 50% (Figuras 4C e D).

**Figura 5:**
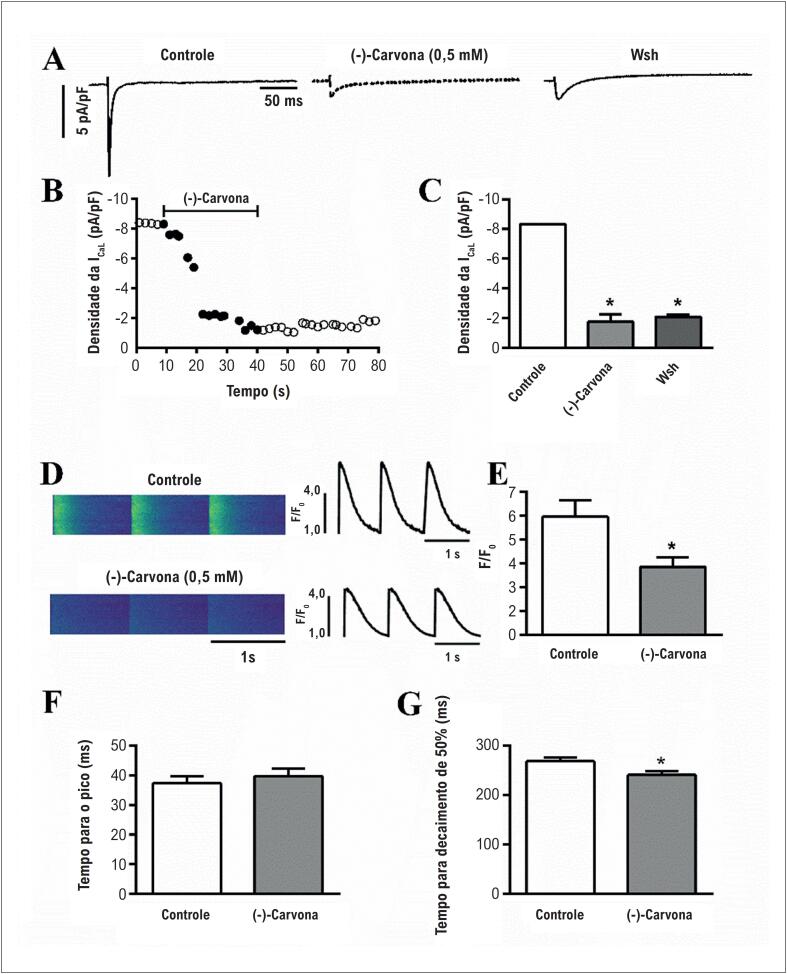
Efeitos da (-)-carvona sobre a corrente de cálcio do tipo L (I_Ca,L_) e transiente de cálcio intracelular no cardiomiócitos ventricular isolado. (A) registros típicos de I_Ca,L_ no controle, durante a perfusão com 0,5 mM (-)-carvona e *washout* (Wsh) efeito da (-)-carvona sobre I_Ca,L_ ao longo do tempo. Cada símbolo indica a amplitude líquida da I_Ca,L_ medida a cada 10 s no potencial de membrana 0mV sob condições controle (círculos abertos), durante a exposição a 0,5mM de (-)-carvona (círculos pretos), a após Wash (círculos abertos); (C) resumo dos efeitos da (-)-carvona sobre a densidade da I_Ca,L_ (pA/pF); (D) imagens (esquerda) e traçados representativos (direita) do transiente de cálcio intracelular no controle (painel superior) e após incubação com 0,5 mM de (-)-carvona (painel inferior), (E) média de pico de transiente de cálcio (F/F_0_); (F) tempo para o pico do transiente e (G) tempo para decaimento de 50% de transiente de cálcio (n=4,5, *p<0,05 vs. controle; # p < 0,05 *vs* (-)-carvona).

Considerando o papel principal dos canais de Ca^+2^ do tipo L no controle da contração cardíaca, usamos o teste de grampeamento de voltagem (técnica de *patch-clamp*) para testar se a (-)-carvona afetaria a I_Ca,L_ nos cardiomiócitos dos ventrículos. A Figura 5A mostra registros de I_Ca,L_ das fases de despolarização (300ms) de -40 a 0mV na situação controle e com 0,5mM de (-)-carvona. A Figura 5B ilustra a progressão da I_Ca,L_ ao longo do tempo, mostrando redução da I_Ca,L_ após incubação com (-)-carvona. A média de redução do pico de I_Ca,L_ induzida pela (-)-carvona foi de 79% (n=4, 10 células, Figura 5C). O efeito de 0,3mM de (-)-carvona sobre a I_Ca,L_ também foi avaliado, e se observou uma redução de 43% na I_Ca,L_ (dados não apresentados). Concluímos que a (-)-carvona inibe os canais de Ca^+2^ do tipo L, e que esse efeito pode contribuir para seu efeito inotrópico negativo evidenciado nos tecidos dos átrios e ventrículos.

Com base nesses resultados, buscamos avaliar o transiente de cálcio intracelular em cardiomiócitos ventriculares imersos com FUO4-AM. A Figura 5D (esquerda) mostra as imagens obtidas usando microscopia confocal do transiente de cálcio intracelular no controle e após pré-incubação com 0,5mM de (-)-carvona. Observou-se que a fluorescência do cálcio, mostrada em verde, foi reduzida com (-)-carvona. A Figura 5D (à direita) mostra traçados representativos do transiente de cálcio intracelular no controle e com (-)-carvona. A Figura 5E apresenta a fluorescência do cálcio como razão F/F_0_, a qual foi reduzida após incubação com (-)-carvona (n=5). O pré-tratamento dos cardiomiócitos com (-)-carvona acelerou o tempo de decaimento em 50% (Figura 5G), enquanto que o tempo para pico do transiente de Ca^+2^ (Figura 5F) não foi alterado.

**Figura 6:**
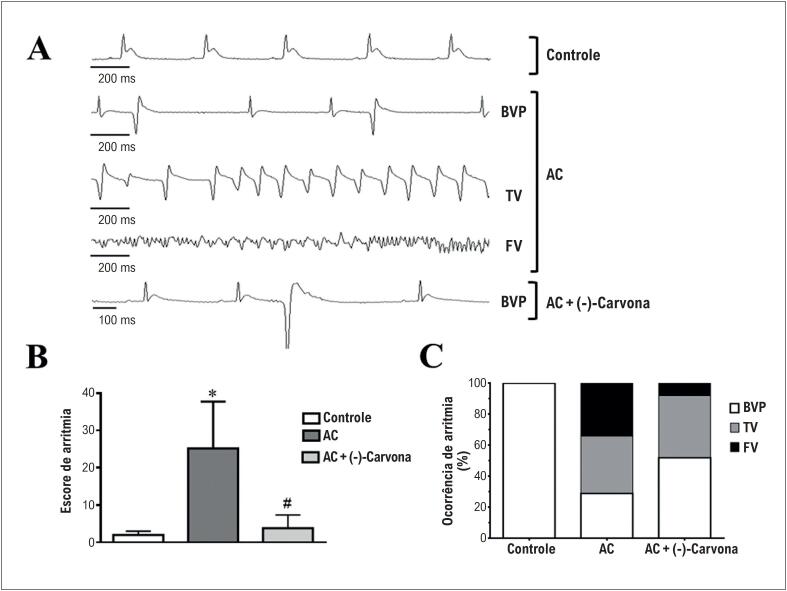
Efeito antiarrítmico da (-)-carvona no modelo de arritmia induzida por sobrecarga de cálcio. (A) eletrocardiogramas representativos no controle, com alta concentração de cálcio (AC) e AC mais (-)-carvona, mostrando as seguintes arritmias: batimentos ventriculares prematuros (BVP), taquicardia ventricular (TV) e e fibrilação ventricular (FV); (B) escore de arritmia e (C) ocorrência de arritmia (n=5, *p<0,05 vs. controle e #p < 0,05 vs AC).

Uma vez que os bloqueadores de canais de cálcio apresentam efeitos antiarrítmicos, decidimos investigar se a (-)carvona apresentaria essa propriedade. O efeito antiarrítmico da (-)-carvona foi avaliado em um modelo de arritmia induzido pela sobrecarga de cálcio. Três tipos de arritmias foram observados nos corações perfundidos com alta concentração de cálcio: BVP, TC e FV (Figura 6A). Como observado na Figura 6B, a (-)-carvona reduziu significativamente o escore de arritmia (n=5). Além disso, nossos resultados mostraram que nos corações submetidos à cálcio elevado e perfusão simultânea com (-)-carvona, a gravidade das arritmias foi menor, uma vez que a ocorrência de FV diminuiu de 34% (cálcio elevado) para 8%. Ainda, os corações perfundidos com (-)-carvona apresentaram principalmente BVP, considerada uma arritmia de menor gravidade.

## Discussão

Nossos resultados mostraram a capacidade do monoterpeno (-)-carvona em reduzir a força atrial de corações de ratos de maneira concentração-dependente, o que foi parcialmente reversível após a lavagem. A (-)-carvona mostrou baixa potência em comparação à nifedipina, um clássico bloqueador de canal de C^+2^ tipo L. Sabe-se que a força contrátil é dependente da concentração de Ca^+2^ citoplasmático livre, e que a entrada de Ca^+2^ através de canais de Ca^+2^ do tipo L é essencial para estimular a liberação de cálcio induzida pelo cálcio do retículo sarcoplasmático (RS). Esse mecanismo é muito importante por regular a força do miocárdio.

Assim, decidimos investigar se havia uma correlação entre a redução da força atrial e uma diminuição na entrada de Ca^+2^ no mecanismo de ação da (-)-carvona. Nossos resultados mostraram que a (-)-carvona reduziu a entrada de Ca^+2^ ao prejudicar a resposta inotrópica positiva tanto para Ca^+2^ como para Bay K 8644, um agonista dos canais de Ca^+2^ do tipo L. O bloqueio do canal de Ca^+2^ promovido pela (-)-carvona foi provavelmente o responsável pela diminuição da força atrial observada em nossos experimentos. Em músculos lisos, a carvona apresenta efeito antiespasmódico; ela reduziu a contração induzida por alta concentração de K^+^ e foi quase 100 vezes mais potente que o verapamil, um bloqueador de canal de cálcio.^13,20^

A capacidade dos terpenos em bloquear o canal de Ca^+2^ foi observada tanto no músculo liso como no músculo cardíaco.^21^ Os monoterpenos podem modular a função dos canais iônicos dependentes de voltagem e dependentes de ligantes.^22,23^ Assim, esses compostos são úteis em prevenir doenças cardiovasculares, tais como arritmia e hipertensão. Em relação ao sistema cardiovascular, monoterpenos tais como rotundifolona,^24^ terpineol,^25^ timol,^23^ e carvacrol^23^ atuam como bloqueadores de canal de cálcio. Também foi demonstrado que em cardiomiócito isolado, R(+)-pulegona,^16^ geraniol,^6^ nerol,^7^ farnesol^9^ e (-)-mentol^26^ bloquearam o canal de Ca^+2^ do tipo L.

O bloqueio dos canais de Ca^+2^ pode induzir alterações eletrofisiológicas importantes, como uma diminuição da condução elétrica no coração e da frequência cardíaca. Assim, nós investigamos se a (-)-carvona poderia induzir alterações fisiológicas no coração. Realizamos experimentos com corações isolados para registrar simultaneamente a PVD e perfis eletrocardiográficos. A (-)-carvona promoveu uma diminuição na PVD, o que corrobora nossos resultados obtidos no átrio esquerdo isolado, discutido anteriormente, bem como uma redução na frequência cardíaca. Como se sabe, a frequência cardíaca é usualmente controlada pelo marcapasso primário do coração, o nó sinusal. As células do nó sinusal têm a propriedade de automaticidade como resultado de despolarização gradual durante a diástole elétrica (despolarização diastólica lenta). Uma lenta despolarização diastólica e a fase da despolarização do potencial de ação do marcapasso são processos fundamentais para a formação de impulso elétrico do nó sinusal. Esses fenômenos estão ligados à entrada de Ca^+2^ pelo sarcolema; uma redução no influxo pode induzir a dissociação eletromecânica do miocárdio e bradicardia.^27^ A corrente iônica provavelmente afetada, e responsável pela diminuição da frequência cardíaca, é a I_Ca,L_. O efeito da (-)-carvona sobre o influxo de cálcio promoveu uma redução na frequência cardíaca e um aumento na duração do intervalo PRi, indicativo de bloqueio atrioventricular de primeiro grau. Nesse bloqueio, há um atraso na transmissão de impulso elétrico dos átrios para os ventrículos, aumentando o período refratário do miocárdio. Outras substâncias que promovem esse bloqueio são betabloqueadores, glicosídeos cardíacos, e drogas que aumentam a atividade colinérgica.^28^

Também foi observado que a (-)-carvona aumentou o intervalo QTc, que reflete o período necessário para a despolarização e a repolarização ventricular ocorrer, *i.e*., um parâmetro indireto para estimar a duração do potencial de ação ventricular. O prolongamento do QTc pode ocorrer devido ao bloqueio dos canais de potássio.^6,9^ Agentes antiarrítmicos da classe III são bloqueadores de canais de potássio que prolongam a duração do potencial de ação aumentando o período refratário dos tecidos atrial, nodal e ventricular. Um aumento no período refratário das células atriais é de grande importância no tratamento de taquiarritmia atrial.^29^ A amiodarona, um bloqueador de múltiplos canais, é considerada uma das drogas antiarrítmicas mais efetivas, sendo amplamente prescrita. Contudo, o uso desses medicamentos em longo prazo tem sido associado à ocorrência de torsades de pointes^30^ e de efeitos adversos.^29^

Em cardiomiócitos ventriculares isolados, 0,5mM de (-)-carvona reduziu a fração de encurtamento e acelerou o tempo de relaxamento, conforme foi também observado no coração isolado. Sabe-se que a força contrátil do músculo cardíaco depende da concentração de Ca^+2^ livre no citoplasma, e a entrada de Ca^+2^ através de canais de Ca^+2^ do tipo L é essencial para a liberação de cálcio dependente de cálcio do RS. Assim, o teste de grampeamento de voltagem foi realizado para testar se a (-)-carvona afeta a I_Ca,L._ Os resultados mostraram que a (-)-carvona reduziu significativamente a I_Ca,L_ no cardiomiócito ventricular. Uma vez que a (-)-carvona reduz a I_Ca,L_, é razoável pensar que esse monoterpeno afetaria profundamente a liberação de Ca^+2^ do RS. Nossos resultados mostraram que a (-)-carvona também afetou a amplitude do transiente de Ca^+2^ e acelerou o tempo de decaimento de 50%. Sabe-se que o relaxamento do músculo cardíaco é amplamente determinado pela recaptação de Ca^+2^ pelo retículo sarco(endo)plasmático Ca^2+^-ATPase (SERCA2a) e por outra proteína de transporte tais como os trocadores de Na+/Ca^2+^ (NCX) e membrana plasmática Ca^2+^ ATPase (PMCA).^17^ Assim, a diminuição do Ca^+2^ citosólico pode estar associada com a ativação de algumas dessas vias.

A nifedipina (10 µM), um bloqueador de canal de cálcio do tipo L, reduziu a amplitude do Ca^+2^ transiente em 79% em miócitos do ventrículo de rato neonatos.^24^ O bloqueio produzido pela nifedipina (1 µM) foi totalmente reversível após o *washout* com solução padrão.^31^ Nossos resultados indicam que a (-)-carvona é um bloqueador de canal de Ca^+2^, similar à nifedipina, mas o efeito sobre a I_Ca,L_ foi irreversível na presença de (-)-carvona (500 μM). Segundo Vaughan-Williams (1970), os bloqueadores de canal de cálcio pertencem à classe IV de antiarrítmicos, e são amplamente utilizados na prática clínica.^32,33^

Uma vez que a (-)-carvona reduz a entrada de cálcio sarcolemal, nós investigamos sua possível atividade antiarrítmica e observamos uma drástica redução ao longo do tempo em eventos como FV em um modelo *ex vivo* de sobrecarga de cálcio. De fato, nossos resultados mostraram que a (-)-carvona apresentou um bom efeito antiarrítmico, confirmado por uma redução nos escores de arritmia e redução na ocorrência de fibrilação atrial, considerada um tipo mais grave de arritmia. Sabe-se que as substâncias ativas oriundas de plantas podem apresentar propriedades antiarrítmicas importantes,^34^ com grande potencial para serem usadas como agentes antiarrítmicos em estudos clínicos e pré-clínicos. Podemos citar, por exemplo, os terpenos geraniol, nerol, D-limoneno e farnesol que inibiram canais de Ca^+2^ do tipo L e apresentaram atividade antiarrítmica.^6–9^ Embora muitos estudos experimentais mostraram que os terpenos exercem efeitos antiarrítmicos, esses compostos não são ainda usados na clínica. Além do efeito antiarrítmico, a (-)-carvona também mostrou um efeito cardioprotetor contra a cardiotoxicidade induzida por doxorrubicina *in vivo* e potencializou sua toxicidade anticâncer *in vitro*.^14^ Esses efeitos cardioprotetores fazem a carvona uma molécula promissora na prática clínica.

### Limitações do estudo

Este estudo revelou que a (-)-carvona reduz a corrente de cálcio do tipo L, induz efeito inotrópico negativo, e tem efeitos antiarrítmicos no coração de ratos. Porém, podemos apontar também algumas limitações, como a falta de avaliação dos efeitos antiarrítmicos da (-)-carvona, em um modelo *in vivo* de arritmia e outros modelos *in vitro* que indiretamente geram uma sobrecarga de cálcio. Outra limitação deste estudo foi o fato de não termos avaliado os efeitos da carvona sobre outros canais importantes para a excitação cardíaca, tampouco sua ação sobre a SERCA2a. Ainda, há outras limitações, incluindo implicações toxicológicas do uso agudo e em longo prazo da carvona, sua metabolização e farmacodinâmica.

## Conclusão

Podemos concluir que a (-)-carvona diminuiu a corrente de cálcio do tipo L e o transiente de cálcio intracelular no miocárdio, promovendo uma redução na contratilidade atrial e ventricular. Em corações de ratos isolados, a (-)-carvona causou uma diminuição nas taxas cardíacas e aumento em intervalos PR, característica de bloqueadores de canais de cálcio. Ainda, observou-se uma redução significativa da gravidade das arritmias, como fibrilação ventricular em corações submetidos à perfusão com carvona. A (-)-carvona é, portanto, uma substância natural altamente promissora em relação ao desenvolvimento de novas drogas antiarrítmicas.

## References

[1] Cronin EM, Bogun FM, Maury P, Peichl P, Chen M, Namboodiri N, et al. 2019 HRS/EHRA/APHRS/LAHRS Expert Consensus Statement on Catheter Ablation of Ventricular Arrhythmias. Heart Rhythm. 2020;17(1):2-154. doi: 10.1016/j.hrthm.2019.03.002. 10.1016/j.hrthm.2019.03.002 PMC8453449 31085023

[2] Fishman GI. Drug-Induced Arrhythmias, Precision Medicine, and Small Data. Circ Arrhythm Electrophysiol. 2017;10(4):e005208. doi: 10.1161/CIRCEP.117.005208. 10.1161/CIRCEP.117.005208 PMC5470633 28408653

[3] Benchimol A, Desser KB. New Drugs for Treating Cardiac Arrhythmias. Postgrad Med. 1981;69(1):77-84. doi: 10.1080/00325481.1981.11715649. 10.1080/00325481.1981.11715649 6780988

[4] Chia KKM, Kanagaratnam L, Hellestrand K, Kowey P, Whalley D. Pharmacological Therapy for Ventricular Arrhythmias: A State-of-the Art Review. Heart Lung Circ. 2019;28(1):49-56. doi: 10.1016/j.hlc.2018.10.002. 10.1016/j.hlc.2018.10.002 30392983

[5] Ganjehei L, Massumi A, Nazeri A, Razavi M. Pharmacologic Management of Arrhythmias. Tex Heart Inst J. 2011;38(4):344-9. PMC3147219 21841856

[6] Menezes-Filho JE, Gondim AN, Cruz JS, Souza AA, Santos JN, Conde-Garcia EA, et al. Geraniol Blocks Calcium and Potassium Channels in the Mammalian Myocardium: Useful Effects to Treat Arrhythmias. Basic Clin Pharmacol Toxicol. 2014;115(6):534-44. doi: 10.1111/bcpt.12274. 10.1111/bcpt.12274 24862086

[7] Menezes-Filho JER, Souza DS, Santos-Miranda A, Cabral VM, Santos JNA, Cruz JDS, et al. Nerol Attenuates Ouabain-Induced Arrhythmias. Evid Based Complement Alternat Med. 2019;2019:5935921. doi: 10.1155/2019/5935921. 10.1155/2019/5935921 PMC6431517 30984275

[8] Nascimento GAD, Souza DS, Lima BS, Vasconcelos CML, Araújo AAS, Durço AO, et al. Bradycardic and Antiarrhythmic Effects of the D-Limonene in Rats. Arq Bras Cardiol. 2019;113(5):925-32. doi: 10.5935/abc.20190173. 10.5935/abc.20190173 PMC7020959 31482987

[9] Souza DS, Menezes-Filho JER, Santos-Miranda A, Jesus ICG, Silva Neto JA, Guatimosim S, et al. Calcium Overload-induced Arrhythmia is Suppressed by Farnesol in Rat Heart. Eur J Pharmacol. 2019;859:172488. doi: 10.1016/j.ejphar.2019.172488. 10.1016/j.ejphar.2019.172488 31233746

[10] Elmastaş M, Dermirtas I, Isildak O, Aboul‐Enein HY. Antioxidant Activity of S‐Carvone Isolated from Spearmint (Mentha Spicata L. Fam Lamiaceae). J. Liq. Chromatogr. Relat. Technol. 2006;29(10):1465-75. doi: 10.1080/10826070600674893

[11] Moro, I. J. et al. Evaluation of antimicrobial, cytotoxic and chemopreventive activities of carvone and its derivatives. Braz. J. Pharm. Sci. 2017;539(4): e00076. doi: 10.1590/s2175-97902017000400076.

[12] Nogoceke FP, Barcaro IM, Sousa DP, Andreatini R. Antimanic-like Effects of (R)-(-)-carvone and (S)-(+)-carvone in Mice. Neurosci Lett. 2016;619:43-8. doi: 10.1016/j.neulet.2016.03.013. 10.1016/j.neulet.2016.03.013 26970377

[13] Souza FV, Rocha MB, Souza DP, Marçal RM. (-)-Carvone: Antispasmodic Effect and Mode of Action. Fitoterapia. 2013;85:20-4. doi: 10.1016/j.fitote.2012.10.012. 10.1016/j.fitote.2012.10.012 23103297

[14] Abbas MM, Kandil Yİ, Abbas MA. R-(-)-carvone Attenuated Doxorubicin Induced Cardiotoxicity In Vivo and Potentiated Its Anticancer Toxicity In Vitro. Balkan Med J. 2020;37(2):98-103. doi: 10.4274/balkanmedj.galenos.2019.2019.7.117. 10.4274/balkanmedj.galenos.2019.2019.7.117 PMC7094179 31893584

[15] Curtis MJ, Alexander S, Cirino G, Docherty JR, George CH, Giembycz MA, et al. Experimental Design and Analysis and their Reporting II: Updated and Simplified Guidance for Authors and Peer Reviewers. Br J Pharmacol. 2018;175(7):987-93. doi: 10.1111/bph.14153. 10.1111/bph.14153 PMC5843711 29520785

[16] Cerqueira SV, Gondim AN, Roman-Campos D, Cruz JS, Passos AG, Lauton-Santos S, et al. R(+)-pulegone Impairs Ca²+ homeostasis and Causes Negative Inotropism in Mammalian Myocardium. Eur J Pharmacol. 2011;672(1-3):135-42. doi: 10.1016/j.ejphar.2011.09.186. 10.1016/j.ejphar.2011.09.186 22004607

[17] Shioya T. A Simple Technique for Isolating Healthy Heart Cells from Mouse Models. J Physiol Sci. 2007;57(6):327-35. doi: 10.2170/physiolsci.RP010107. 10.2170/physiolsci.RP010107 17980092

[18] Zhou P, Zhang SM, Wang QL, Wu Q, Chen M, Pei JM. Anti-arrhythmic Effect of Verapamil is Accompanied by Preservation of cx43 Protein in Rat Heart. PLoS One. 2013;8(8):e71567. doi: 10.1371/journal.pone.0071567. 10.1371/journal.pone.0071567 PMC3741134 23951191

[19] Curtis MJ, Walker MJ. Quantification of Arrhythmias Using Scoring Systems: an Examination of Seven Scores in an In Vivo Model of Regional Myocardial Ischaemia. Cardiovasc Res. 1988;22(9):656-65. doi: 10.1093/cvr/22.9.656. 10.1093/cvr/22.9.656 3242835

[20] Bers DM. Cardiac Excitation-contraction Coupling. Nature. 2002;415(6868):198-205. doi: 10.1038/415198a. 10.1038/415198a 11805843

[21] Silva-Filho JC, Oliveira NN, Arcanjo DD, Quintans-Júnior LJ, Cavalcanti SC, Santos MR, et al. Investigation of Mechanisms Involved in (-)-borneol-induced Vasorelaxant Response on Rat Thoracic Aorta. Basic Clin Pharmacol Toxicol. 2012;110(2):171-7. doi: 10.1111/j.1742-7843.2011.00784.x. 10.1111/j.1742-7843.2011.00784.x 21883938

[22] Oz M, El Nebrisi EG, Yang KS, Howarth FC, Al Kury LT. Cellular and Molecular Targets of Menthol Actions. Front Pharmacol. 2017;8:472. doi: 10.3389/fphar.2017.00472. 10.3389/fphar.2017.00472 PMC5513973 28769802

[23] Peixoto-Neves D, Silva-Alves KS, Gomes MD, Lima FC, Lahlou S, Magalhães PJ, et al. Vasorelaxant Effects of the Monoterpenic Phenol Isomers, Carvacrol and Thymol, on Rat Isolated Aorta. Fundam Clin Pharmacol. 2010;24(3):341-50. doi: 10.1111/j.1472-8206.2009.00768.x. 10.1111/j.1472-8206.2009.00768.x 19682086

[24] Guedes DN, Silva DF, Barbosa-Filho JM, Medeiros IA. Calcium Antagonism and the Vasorelaxation of the Rat Aorta Induced by Rotundifolone. Braz J Med Biol Res. 2004;37(12):1881-7. doi: 10.1590/s0100-879x2004001200014. 10.1590/s0100-879x2004001200014 15558195

[25] Khaleel C, Tabanca N, Buchbauer G. α-Terpineol, a Natural Monoterpene: A Review of its Biological Properties. Open Chem. 2018;16(1):349–61. doi: 10.1515/chem-2018-0040.

[26] Baylie RL, Cheng H, Langton PD, James AF. Inhibition of the Cardiac L-type Calcium Channel Current by the TRPM8 Agonist, (-)-menthol. J Physiol Pharmacol. 2010;61(5):543-50. 21081797

[27] Lipsius SL, Hüser J, Blatter LA. Intracellular Ca2+ Release Sparks Atrial Pacemaker Activity. News Physiol Sci. 2001;16:101-6. doi: 10.1152/physiologyonline.2001.16.3.101. 10.1152/physiologyonline.2001.16.3.101 11443225

[28] Zhou Q, Xiao J, Jiang D, Wang R, Vembaiyan K, Wang A, et al. Carvedilol and its New Analogs Suppress Arrhythmogenic Store Overload-induced Ca2+ Release. Nat Med. 2011;17(8):1003-9. doi: 10.1038/nm.2406. 10.1038/nm.2406 PMC3268079 21743453

[29] Mehraein, F. A Review on Amiodarone as an Antiarrhythmic Drug. Abnorm. Heart Rhythms. 2015; 96:1593-600. doi: 10.1136/hrt.2008.152652.

[30] Tisdale JE. Drug-induced QT Interval Prolongation and Torsades de Pointes: Role of the Pharmacist in Risk Assessment, Prevention and Management. Can Pharm J (Ott). 2016;149(3):139-52. doi: 10.1177/1715163516641136. 10.1177/1715163516641136 PMC4860751 27212965

[31] Wang F, Koide M, Wellman GC. Nifedipine Inhibition of High-Voltage Activated Calcium Channel Currents in Cerebral Artery Myocytes Is Influenced by Extracellular Divalent Cations. Front Physiol. 2017;8:210. doi: 10.3389/fphys.2017.00210. 10.3389/fphys.2017.00210 PMC5383720 28439241

[32] Gao H, Wang F, Wang W, Makarewich CA, Zhang H, Kubo H, et al. Ca(2+) Influx through L-type Ca(2+) Channels and Transient Receptor Potential Channels Activates Pathological Hypertrophy Signaling. J Mol Cell Cardiol. 2012;53(5):657-67. doi: 10.1016/j.yjmcc.2012.08.005. 10.1016/j.yjmcc.2012.08.005 PMC3472041 22921230

[33] Williams EMV. A Classification of Antiarrhythmic Actions Reassessed After a Decade of New Drugs. J Clin Pharmacol. 1984;24(4):129-47. doi: 10.1002/j.1552-4604.1984.tb01822.x. 10.1002/j.1552-4604.1984.tb01822.x 6144698

[34] Bryzgalov AO, Tolstikova TG, Shults EE, Petrova KO. Natural Products as a Source of Antiarrhythmic Drugs. Mini Rev Med Chem. 2018;18(4):345-62. doi: 10.2174/1389557516666161104144815. 10.2174/1389557516666161104144815 27823558

