# Supplementary material for: A (-)-Carvona Modula a Sinalização de Cálcio Intracelular com Ação Antiarrítmica em Corações de Ratos
Source: Arq Bras Cardiol. 2022 Jul 29;119(2):294–304. [Article in Portuguese] doi: 10.36660/abc.20210499 (PMC9363060; doi:10.36660/abc.20210499)
Supplement: Translation

# Translation: (-)-Carvone Modulates Intracellular Calcium Signaling with Antiarrhythmic Action in Rat Hearts

**Authors:** Gilmara Beatriz Andrade da Silva, Diego Santos Souza, José Evaldo Rodrigues Menezes-Filho, Júlio Alves da Silva-Neto, Jader dos Santos Cruz, Danilo R. Roman-Campos, Lucindo José Quintans-Júnior, Carla Maria Lins de Vasconcelos

**Affiliations:** 1 São Cristóvão SE Brazil Universidade Federal de Sergipe, São Cristóvão, SE – Brazil; 2 Belo Horizonte MG Brazil Universidade Federal de Minas Gerais – Instituto de Ciências Biológicas, Belo Horizonte, MG – Brazil; 3 São Paulo SP Brazil Universidade Federal de São Paulo, São Paulo, SP – Brazil

**Keywords:** Arrhythmias, Cardiac, Monoterpenes, Rats

## Abstract

**Background:**

(-)-Carvone is a monoterpene found in essential oils with antioxidant and anti-inflammatory activity.

**Objective:**

The aim of this paper was to analyze the antiarrhythmic property of (-)-carvone in the rat heart and its effects on the intracellular Ca^2+^ signaling.

**Methods:**

The effects of (-)-carvone were evaluated on the ventricular (0.5 mM) and atrial contractility (0.01 – 4 mM) and on electrocardiogram (0.5 mM). Fractional shortening, L-type calcium current (I_Ca,L_) and Ca^2+^ signaling were measured in the isolated cardiomyocyte (0.5 mM). Antiarrhythmic effect was evaluated in arrhythmia model induced by calcium overload (0.5 mM) (n = 5). P < 0.05 was used as the significance level.

**Results:**

In the atrium, (-)-carvone evoked negative inotropism that was concentration-dependent (EC50 0.44 ± 0.11 mM) and decreased the positive inotropism evoked by CaCl_2_ (0.1 to 8.0 mM) or BAY K8644 (5 to 500 nM), an agonist of L-type Ca^2+^ channel. In isolated heart, (-)-carvone (0.5 mM) promoted reduction of ventricular contractility (73%) and heart rate (46%), increased PRi (30.7%, time from the onset of the P wave until the R wave) and QTc (9.2%, a measure of the depolarization and repolarization of the ventricles) without changing the QRS complex duration. (-)-Carvone decreased the fractional shortening (61%), I_Ca,L_ (79%) and Ca^2+^ intracellular transient (38%). Furthermore, (-)-carvone showed antiarrhythmic action, verified by decrease of the arrhythmia score (85%) and occurrence of ventricular fibrillation.

**Conclusion:**

(-)-Carvone decreases Ca^2+^ entry through L-type Ca^2+^ channels, reducing the cardiac contractility and intracellular Ca^2+^, and, therefore, presenting promising antiarrhythmic activity in the rat hearts.

## Introduction

Arrhythmias are considered a serious public health problem and are an important cause of morbidity and mortality in the world.^1^ Among the main cardiac arrhythmias, ventricular premature beats (VPB), sustained ventricular tachycardia and fibrillation are common in patients with ischemic and nonischemic cardiomyopathy.^1^ However, treatments with antiarrhythmic drugs often cause pro-arrhythmic adverse responses or no improvement in the quality of life of people affected by arrhythmias.^2^

Since 1970, when Vaughan-Williams classified the antiarrhythmic drugs based on their pharmacological mechanisms to block specific ion channels or receptors, researchers have invested a great deal of time and effort to discover new therapies with a lower risk of adverse effects for the patient.^3–5^

Among the new therapies, compounds of natural origin have demonstrated their capacity to inhibit ventricular cardiac arrhythmias generating interest in the scientific community. Terpenes have been shown to be the main compounds with proven antiarrhythmic activity.^6–9^ Among these terpenes, one of particular interest is carvone (*p*-mentha-6,8-dien-2-one) because of its already established properties. Carvone is a monoterpene ketone known for its antioxidant, antimicrobial and antifungal activity.^10,11^ It has also been reported to have a blocking effect on voltage-gated sodium channels in neurons, leading to an anticonvulsant effect.^12,13^ Furthermore, carvone has also shown an antispasmodic effect through voltage-dependent calcium channel inhibition, and synergistic anticancer action with doxorubicin on the MCF 7 cell line while decreasing its cardiotoxicity.^14^ As has already been described in the literature, carvone blocks sodium and calcium channels, and drugs of this class have antiarrhythmic and cardioprotective properties. Therefore, we decided to study the effects or carvone on the cellular calcium handling and its possible antiarrhythmic action in rat hearts.^9^

Although there are several studies about (-)-carvone in the scientific literature, there are, to the best of our knowledge, no explanations or hypotheses on the mechanism of action of this monoterpene in the cardiac muscle. Therefore, our objective was to assess the possible cardiac effects of (-)-carvone, and to provide a better scientific understanding of its action in cardiac tissue that might serve as basis for the development of new drugs of natural origin for the treatment of arrhythmias.

## Material and Methods

### Animals

The experiments were performed using male Wistar rats (250-300 g) obtained from the animal care facility of the Federal University of Sergipe (UFS). In each experimental procedure, five animals were used.^15^ This research was approved by the Ethics Committee on Animal Research of the UFS (protocol 61/16, February 20^th^ 2017). Animal handling was in compliance with the Principles of Laboratory Animal Care (NIH publication 86-23, revised 1985; http://oacu.od.nih.gov/regs/index.htm).

### Evaluation of inotropic effect of (-)-carvone

The inotropic effect of (-)-carvone was evaluated in the left atrium of rat hearts immersed in an organ bath containing Krebs-Henseleit solution comprising (in mM): NaCl 120, KCl 5.4, MgCl_2_ 1.2, NaHCO_3_ 27, CaCl_2_ 1.25, Glucose 10, NaH_2_PO_4_ 2.0 (pH 7.4). The atrium was maintained at 29° ± 0.1°C, oxygenated (95% O_2_ and 5% CO_2_), stretched to 5 mN and submitted to field stimulation (1 Hz, 100 V, 0.5 ms) (Stimulator SD9 GRASS). Atrial force was recorded using an isometric force transducer (GRASS FT03), and the signals digitalized (DATAQ DI710, WINDAQ PRO Acquisition). The concentration-response curves of (-)-carvone (0.001 to 4.0 mM) and nifedipine (0.03 to 100 µM, Ca^2+^ channel blocker) were obtained to determine contractile response and calculate the EC_50_. Dimethyl sulfoxide (DMSO) at 0.5% was used as the diluent for (-)-carvone.

#### Effects of (-)-carvone on Ca^2+^ influx in the atrial myocardium

To analyze the effect of (-)-carvone on the Ca^2+^ influx, the concentration-response curves of CaCl_2_ (0.1 to 8.0 mM) and (±)-Bay K8644 (5 to 500 nM) in the left atrium in the control and after pre-incubation with (-)-carvone (1 mM) for 15 min were obtained. The results were expressed as percentages of the maximum atrial contractile response to CaCl_2_ in the control. In both protocols, the initial concentration of CaCl_2_ in the K-H solution was 0.5 mM.^7,16^

#### Effects of (-)-carvone on the electrocardiographic profile and left ventricular developed pressure (LVDP)

After intraperitoneal administration of heparin in rats (1000 IU) for 15 minutes, the hearts were removed and mounted on a constant-flow (10 mL/min) aortic perfusion system. The heart was perfused with previously filtered K-H solution (0.45 µm), oxygenated (95% O_2_ + 5% CO_2_) and maintained at 34 ± 0.1°C (Haake F3). To record the electrocardiogram (ECG), three electrodes (Ag/AgCl/NaCl 1 M) were placed on the heart to sense electrical signals. The signals were amplified and digitalized (PowerLab 4/35 ADInstrument, USA). LVDP was measured using a water-filled balloon (15 cm/Hg) introduced into the cavity of the left ventricle. This device was coupled to a pressure transducer (MLT0699/A). The signals were amplified (Bridge Amp FE221 ADInstrument, USA) and sent to an AD converter (PowerLab 4/35 26 ADInstrument, USA). The system was calibrated using a column of mercury. Contractile parameters (LVDP, time to peak and relaxation time) were evaluated in 30 consecutive beats using LabChart 8.0 Pro Software (ADInstruments, USA) in control situation and after 5, 10 and 15 minutes from the start of carvone perfusion (0.5 mM). The ECG measured the PR interval (PRi - the period that extends from the beginning of the P wave until the R wave), QRS complex duration (QRS - the period that extends from the Q wave until the S wave), and the QT interval (QTi - the period that extends from the beginning of the Q until the end of the T wave). QTi was converted to QTc using the Bazett’s formula normalized for rodents (QTc-B = QTi/*RR*/*f*), f is the average duration of RR interval in control (f = 271 ms).

#### Effects of (-)-carvone on the fractional shortening

Left and right ventricular cardiomyocytes were isolated from rats according to the protocol of Shioya (2007),^17^ with some modifications. Shortening fraction was assessed by measuring the change in cell length using an inverted microscope coupled to an edge detection system (Ionoptix, USA). The cardiomyocytes were placed in an experimental chamber (room temperature) containing Tyrode solution (in mM: NaCl 150, KCl 5.4, MgCl_2_ 0.5, HEPES 10, Glucose 10, CaCl_2_ 1.8, pH 7.4). The cardiomyocytes were visualized using a camera (Ionoptix Myocam at 240 Hz) coupled to a microscope and an image detection program (Ionoptix Ionwizard 6.3) was used. Cells were submitted to an electrical field (1 Hz, 100 V, 4 ms) using a pair of platinum electrodes. The longitudinal changes in the borders of the cardiomyocytes were captured by the edge detection system and the generated data were stored and analyzed. The fractional shortening was evaluated in control cells and after incubation with 0.5 mM (-)-carvone.

#### Effects of (-)-carvone on the L-type calcium current (I_Ca,L_)

Whole-cell voltage-clamp recordings were obtained using an EPC 10.2 (HEK Elektronik, Germany). In whole-cell configuration, 3-5 min was waited to establishment of an ionic equilibrium between the pipette solution and intracellular environment. The recording electrodes had tip resistances of 2-3 MΩ. Ventricular cardiomyocytes with series resistance above 8 MΩ were discarded. The composition of internal solution was (in mM): 120 CsCl, 20 TEACl, 5 NaCl, 10 HEPES and 10 EGTA, 1 MgCl_2_ (pH was set to 7.2 using CsOH) and external solution was (in mM): 150 TEACl, 0.5 MgCl_2_, 1.8 CaCl_2_, 10 HEPES and 11 glucose (pH 7.4 set using TEAOH). To evaluate the acute effects of 0.3 and 0.5 mM (-)-carvone on I_Ca,L_, a time course of I_Ca,L_ peak current was recorded in absence and after exposure to a given concentration of (-)-carvone. Pre-pulses from a holding potential of −80 mV to −40 mV for 50 ms was applied to inactivate any remnant Na^+^ or T-type Ca^2+^ channels. Then, a test pulse to 0 mV was applied during 300 ms to measure I_Ca,L_.

#### Effects of (-)-carvone on the intracellular global Ca^2+^ transient

Left and right ventricular cardiomyocytes were loaded with 10 M of FLUO4-AM (Molecular Probes, Eugene, OR, USA) diluted in DMSO for 30 min. To remove the excess dye the cells were washed with Tyrode solution (1.8 mM Ca^2+^). A confocal system (LSM 510 Meta, Zeiss GmbH, Jena, Germany) with a 63x oil immersion objective was used for confocal fluorescence imaging. FLUO4-AM was excited at 488 nm (Argon laser) and the emission intensity was measured at 510 nm. Cardiomyocytes were scanned with a 512-pixel line that was positioned along the longitudinal axis of the cell, every 1.54 ms. Digital image processing was performed using IDL programming language (Research Systems, Boulder, CO, USA).^9^ The intracellular Ca^2+^ levels were reported as F/F_0_, where F_0_ is the resting fluorescence. Intracellular global Ca^2+^ transient was recorded in the control and after three minutes of incubation with 0.5 mM (-)-carvone in room temperature.

#### Effects antiarrhythmic of (-)-carvone

Ex vivo arrhythmia was determined in isolated hearts as previously described.^18^ Initially, the hearts were perfused with K-H solution containing 1.25 mM of calcium (control group). After 20 min, the hearts were perfused with K-H solution containing 3.3 mM of calcium (high calcium group) or with high calcium + 0.5 mM (-)-carvone during 15 min (high calcium group + carvone). The ECG was monitored for 15 min to evaluate the occurrence of arrhythmias. The arrhythmias observed were VPB, ventricular tachycardia (VT) and ventricular fibrillation (VF). The 15 minutes of the experiment was divided into three-minute intervals and the arrhythmia scores were added at the end as described by Curtis and Walker (1988).^9,19^ Episodes of VPB < 10 events/3 min were classifed as score 0 and > 10 events/3 min scored 1; 1-5 episodes of VT < 40 s were 2 and > 5 episodes of VT or 1 episode of VF with duration < 40 s were scored 3; 2 - 5 episodes of VT or VF with duration < 80 s were scored 4; > 5 episodes of VF, VT and/or VF with duration < 160 s was scored as 5; VT and/or VF with duration < 300 s was scored as 6 and > 300 s scored as 5.

### Statistical analysis

All results are shown as the means ± standard deviation of mean (S.D). GraphPad Prism v.5.0 (GraphPad Software, CA, USA) was used for the statistical analyses. Data were tested for normality using the Shapiro-Wilk test. Mean values were compared using the one-way analysis of variance (ANOVA) followed by Tukey’s post hoc test or unpaired t-test. P < 0.05 was used as the significance level.

## Results

(-)-Carvone (0.003 to 4 mM) decreased the atrial force in a concentration-dependent manner. Figure 1A shows tracing of curves of isolated atrial contraction in the control situation and with 0.3, 2 and 4 mM of (-)-carvone and washout. As can be seen, 4 mM of (-)-carvone decreased myocardial contractility by approximately 96% and the reversibility after washout was of approximately 65%. Figure 1B shows a concentration-response curve of the negative inotropic effect of (-)-carvone that presented EC_50_ of 0.44 ± 0.11 mM (n = 5). Nifedipine, used as positive control, presented EC_50_ values of 0.0034 ± 0.0011 mM (n = 5). DMSO at 0.5%, used as a diluent, had no effect on atrial force (data not shown).

**Figure 1:** (-)-Carvone exhibited negative inotropic effect and decreased the calcium influx in the rat left atrium. (A) Experimental tracing of isolated atrial contraction in the control, after incubation with (-)-carvone (0.3, 2 and 4 mM) and washout (Wsh); (B) Concentration-response curves of negative inotropism of (-)-carvone and nifedipine (calcium channel blocker), (C) and (D) Concentration-response curves of CaCl_2_ and (±)-BAY K8644 in the absence and presence of 1 mM of (-)-carvone, respectively (n = 5).

As (-)-carvone evoked a negative inotropic effect, we decided to investigate the involvement of calcium channel in its action mechanism. The results revealed that (-)-carvone (1 mM), shifted the concentration-response curve of CaCl_2_ to the right, increasing the EC_50_ of CaCl_2_ from 1.46 ± 0.14 mM (control) to 3.17 ± 0.22 mM (CaCl_2_ + carvone) (Fig. 1C, n = 5, p < 0.05). Interestingly, (-)-carvone impaired the positive inotropism induced by (±)-BAY K8644, an agonist of the L-type calcium channel (Figure 1D).

In the isolated hearts, 0.5 mM (-)-carvone also induced reduction in LVDP, as can be seen in the representative traces shown in Figure 2A (n = 5). A 73% reduction in LVDP was observed after 15 minutes of heart perfusion with 0.5 mM of (-)-carvone (Figure 2B). (-)-Carvone did not change the time to peak (Figure 2C) but it did significantly reduce the relaxation time (24%) after 15 min of (-)-carvone perfusion (Figure 2D).

**Figure 2:** Effects of (-)-carvone on the cardiac contractility in the isolated rat heart. (A) Records of left ventricle developed pressure (LVDP) in the control (top panel) and with 0.5 mM (-)-carvone (bottom panel); (B) LVDP; (C) time to peak; and (D) relaxation time (n = 5, *p < 0.05).

Figure 3A shows representative ECG tracings in the control situation, after 15 min perfusion with 0.5 mM of (-)-carvone and washout. As can be seen, (-)-carvone decreased heart rate (n = 5, Figure 3B) and increased PRi and QTi (n = 5, Figures 3C and D), without changing the duration of QRS complex (Figure 3E).

**Figure 3:** Effects of (-)-carvone on the electrocardiographic profile in the isolated rat heart. (A) Electrocardiogram records in control, with 0.5 mM (-)-carvone as washout (Wsh), (B) Heart rate, (C) PR interval (PRi), (D) QTc interval and (E) QRS complex duration (n = 5, *p < 0.05 vs control and #p < 0.05 vs (-)-carvone).

Figure 4A shows representative records of the cellular contractility in the control situation (top panel) and after perfusion with 0.5 mM of (-)-carvone (bottom panel), showing the reduction in fractional shortening in cardiomyocytes. The average results showed reduction of the fractional shortening after incubation with (-)-carvone (n = 5, Figure 4B). Furthermore, (-)-carvone decreased both time to peak and time to 50% relaxation (Figures 4C and D).

**Figure 4:** Effects of (-)-carvone on fractional shortening in the isolated ventricular cardiomyocyte. (A) Recording of cell shortening in control (top panel) and after incubation with 0.5 mM of (-)-carvone (bottom panel), (B) Fractional shortening in control and (-)-carvone, (C) Time to peak, (D) Time to 50% of relaxation (n = 5, *p < 0.05).

Considering the major role of L-type Ca^2+^ channels in the control of the cardiac contraction, we used whole-cell patch-clamp to test whether (-)-carvone affects I_Ca,L_ in ventricular cardiomyocytes. Figure 5A shows I_Ca,L_ recordings of 300-ms depolarizing steps from −40 to 0 mV in the control situation and with 0.5 mM (-)-carvone. Figure 5B ilustrates the time course of I_Ca,L_ showing reduction of I_Ca,L_ after (-)-carvone incubation. The average decrease of peak I_Ca,L_ induced by (-)-carvone was 79% (n = 4, 10 cells, Fig. 5C). The effect of 0.3 mM of (-)-carvone on the I_Ca,L_ was also evaluated, and a 43% reduction in the I_Ca,L_ was observed (data not shown). We concluded that (-)-carvone inhibits L-type Ca^2+^ channels and that this effect may contribute to its negative inotropic effect evidenced in atrial and ventricle tissues.

**Figure 5:** Effects of (-)-carvone on the L-type calcium current (I_Ca,L_) and intracellular calcium transient in the isolated ventricular cardiomyocyte. (A) Typical recordings of I_Ca,L_ in control, during the perfusion with 0.5 mM (-)-carvone and washout (Wsh), (B) Time course of the effect of (-)-carvone on I_Ca,L_. Each symbol indicates the net amplitude of I_Ca,L_ measured every 10 s at 0 mV membrane potential under control conditions (open circles), during exposure to 0.5 mM (-)-carvone (black circles), and after Wsh (open circles), (C) Summary of the effects of (-)-carvone on the I_Ca,L_ density (pA/pF), (D) Images (left) and representative tracing (right) of the intracellular calcium transient in control (top panel) and after incubation with 0.5 mM of (-)-carvone (bottom panel), (E) Average calcium transient peak (F/F_0_), (F) Time to peak of transient and (G) Time to 50% decay of calcium transient (n = 4-5, *p < 0.05 *vs* control; # p < 0.05 *vs* (-)-carvone).

In view of these results, we sought to evaluate the intracellular calcium transient in ventricular cardiomyocyte loaded with FUO4-AM. Figure 5D (left) shows the images obtained using confocal microscopy of the intracellular calcium transient in the control and after pre-incubation with 0.5 mM of (-)-carvone. It was noted that the fluorescence of calcium, shown in green, was reduced with (-)-carvone. Figure 5D (right) shows representative tracings of the intracellular calcium transient in the control and with (-)-carvone. Figure 5E shows calcium fluorescence as the F/F_0_ ratio, which was reduced after incubation with (-)-carvone (n = 5). (-)-Carvone pretreatment of cardiomyocytes accelerated the time to 50% decay (Figure 5G), whereas the time to peak Ca^2+^ transient (Fig. 5F) remained unchanged.

Since calcium channel blocking drugs present antiarrhythmic effects, we decided to investigate whether (-)-carvone could present this property. The antiarrhythmic effect of (-)-carvone was evaluated in an arrhythmia model induced by calcium overload. Three types of arrhythmias were observed in hearts perfused with high calcium: VPB, VT, e VF (Figure 6A). As can be seen in Figure 6B, (-)-carvone significantly decreased the arrhythmia score (n = 5). In addition, our results showed that in hearts subjected to high calcium with simultaneous perfusion of (-)-carvone the severity of arrhythmias was lower, as the occurrence of VF decreased from 34% (high calcium) to 8%. Furthermore, the hearts perfused with (-)-carvone had mostly VPB (52%), considered an arrhythmia of lesser severity.

**Figure 6:** Antiarrhythmic effect of (-)-carvone in the calcium overload-induced arrhythmia model. (A) Representative electrocardiograms in control, with high calcium (HC) and HC plus (-)-carvone identifying the arrhythmias: ventricular premature beat (VPB), ventricular tachycardia (VT) and ventricular fibrillation (VF), (B) Arrhythmia score and (C) Occurrence of arrhythmia (n = 5, *p < 0.05 vs control and #p < 0.05 vs HC).

## Discussion

Our results showed the ability of monoterpene (-)-carvone to reduce the atrial force of rat hearts in a concentration-dependent manner that was partially reversible after washing. (-)-Carvone showed low potency when compared to nifedipine, a classic L-type Ca^2+^ channel blocker. It is known that contractile force is dependent on free cytoplasmic Ca^2+^ concentration and that Ca^2+^ influx through L-type Ca^2+^ channels is essential to trigger the calcium-induced calcium release from the sarcoplasmic reticulum (SR).^20^ This mechanism is very important because it regulates myocardial force.

Thus, we decided to investigate whether there was a correlation between atrial force reduction and a decrease in Ca^2+^ entry in the action mechanism of (-)-carvone. Our results showed that (-)-carvone reduced Ca^2+^ influx in the atria by impairing the positive inotropic response to both Ca^2+^ and (-)-Bay K 8644, an agonist of L-type Ca^2+^ channels. The blockade of Ca^2+^ channel promoted by (-)-carvone was probably responsible for the decreased atrial force observed in our experiments. In smooth muscles, carvone presents antispasmodic effect; it reduced the contraction induced by high K^+^ and was almost 100 times more potent than verapamil, a calcium channel blocker.^13^

The ability of terpenes to block the Ca^2+^ channel has been observed both in smooth muscle and cardiac muscle.^21^ Monoterpenes can modulate the function of voltage-dependent and ligand-dependent ion channels.^22,23^ Therefore, they are useful in preventing cardiovascular diseases such as arrhythmia and hypertension. With respect to the cardiovascular system, several studies have reported that monoterpenes such as rotundifolone,^24^ terpineol,^25^ timol,^23^ and carvacrol^23^ act as blockers of calcium channel. It has also been shown that in isolated cardiomyocyte, R(+)-pulegone,^16^ geraniol,^6^ nerol,^7^ farnesol ^9^ and (-)-menthol^26^ blocked the L-type Ca^2+^ channel.

Blockage of Ca^2+^ channels can induce important electrophysiological changes, such as a decrease in electrical conduction in the heart and heart rate. Therefore, we investigated whether (-)-carvone could induce physiological changes in the heart. Experiments using isolated hearts were performed to simultaneously record LVDP and ECG profiles. (-)-Carvone promoted a decrease in LVDP, which corroborates our findings in the isolated left atrium, as discussed earlier, and also induced a reduction in heart rate. As is known, heart rate is usually controlled by the heart’s primary pacemaker, the sinus node. Sinus node cells display the property of automaticity as a result of gradual depolarization during electrical diastole (slow diastolic depolarization). The slow diastolic depolarization and the phase of depolarization of the pacemaker action potential are essential processes for the formation of the electrical impulse of the sinus node. These phenomena are linked to Ca^2+^ influx by the sarcolemma; a decreased influx can induce electromechanical decoupling of the myocardium and bradycardia.^27^ The ionic current that was probably affected, and was responsible for decreasing heart rate, may be the I_Ca,L_. The effect of (-)-carvone on calcium influx promoted a reduction in heart rate and an increase in the duration of the PRi interval, indicative of first-degree atrioventricular block. In this blockage, there is a delay in the transmission of electrical impulse from the atria to the ventricles, increasing the refractory period of myocardium. Other substances that promote this blockage are β-blockers, cardiac glycosides, and drugs that increase cholinergic activity.^28^

It was observed that (-)-carvone also increased the QTc interval, which reflects the period necessary for depolarization and ventricular repolarization to occur, i.e., an indirect parameter to estimate the ventricular action potential duration. QTc prolongation may be due to the blockage of potassium channels.^6,9^ Class III antiarrhythmic agents are potassium channel blockers that prolong the duration of the action potential increasing the refractory period of atrial, nodal and ventricular tissues. An increase in the refractory period of the atrial cells is of great importance in the treatment of atrial tachyarrhythmia.^29^ Amiodarone, a multiple-channel blocker, is considered one of the most effective antiarrhythmic drugs, and is widely prescribed. However, long-term use of antiarrhythmic drugs has been reported to cause torsades de pointes^30^ and adverse effects.^29^

In isolated ventricular cardiomyocytes, 0.5 mM of (-)-carvone reduced the shortening fraction and accelerated the relaxation time, as was also observed in the isolated heart. It is known that the contractile force of the heart muscle depends on the free cytoplasmic Ca^2+^ concentration, and Ca^2+^ influx through L-type Ca^2+^ channels is essential to trigger calcium-induced calcium release from the SR. Then, whole-cell patch-clamp was performed to test whether (-)-carvone affects I_Ca,L_. The results showed that (-)-carvone significantly reduced the I_Ca,L_ in ventricular cardiomyocyte. As (-)-carvone reduces I_Ca,L,_ it is reasonable to think that this monoterpene would profoundly affect the Ca^2+^ release from the SR. Our results showed that (-)-carvone also affected the Ca^2+^ transient amplitude and accelerated the time to 50% decay. It is known that the cardiac muscle relaxation is largely determined by reuptake of Ca^2+^ into the SR by sarco(endo)plasmic reticulum Ca^2+^ ATPase (SERCA2a) and by another transport protein such as Na^+^/Ca^2+^ exchanger (NCX) and plasma membrane Ca^2+^ ATPase (PMCA).^17^ Then, the decrease of the cytosolic Ca^2+^ may be associated with the activation of some of these pathways.

Nifedipine (10 µM), a L-type calcium channel blocker, reduced the amplitude of the Ca^2+^ transient by 79% in neonatal rat ventricular myocytes.^24^ The blockade produced by nifedipine (1 μM) was totally reversible after washout with standard solution.^31^ Our results indicate that (-)-carvone is a Ca^2+^ channel blocker, similar to nifedipine, but the effect on the I_Ca,L_ was irreversible in the presence of 500 μM (-)-carvone. According to Vaughan-Williams (1970), calcium channel blockers belong to class IV of the antiarrhythmics, and are widely used in clinical medicine.^32,33^

As (-)-carvone reduced sarcolemal Ca^2+^ influx, we investigated their possible antiarrhythmic activity and observed a drastic reduction over time in events such as VF in an ex vivo model of calcium overload. Indeed, our results showed that (-)-carvone had a good antiarrhythmic effect, confirmed by decreased arrhythmia scores and reduction in the occurrence of ventricular fibrillation, considered a more severe type of arrhythmia. It is already known that active substances from plant material can have significant antiarrhythmic properties,^34^ with great potential to be used as antiarrhythmics in preclinical and clinical studies. We can cite as example the terpenes geraniol, nerol, D-limonene and farnesol that have been shown to inhibit L-type Ca^2+^ channels and present antiarrhythmic activity.^6–9^ Although many experimental studies have shown that terpenes exert antiarrhythmic effects, these compounds are not yet used in the clinic. In addition to the antiarrhythmic effect, (-)-carvone also had a cardioprotective effect against doxorubicin-induced cardiotoxicity *in vivo* and potentiated its anticancer toxicity *in* vitro.^14^ These cardioprotective effects of carvone make it a promising molecule for use in clinical practice.

### Study limitation

This study revealed that (-)-carvone reduces L-type calcium current, induces negative inotropic effect, and has antiarrhythmic effects in rat hearts. But we also can point some limitations, as the lack of evaluation of antiarrhythmic effects of (-)-carvone in an *in vivo* model of arrhythmia and other *in vitro* models that indirectly generate calcium overload. Another limitation of this study is that we did not assess the effects of carvone on other important channels for cardiac excitation, nor evaluate its action on the SERCA2a. Furthermore, there are other limitations, including toxicological implications of the acute and long-term use of carvone, its metabolization, and pharmacodynamics.

## Conclusion

We can conclude that (-)-carvone decreased L-type calcium current and intracellular calcium transient in the myocardium, promoting a reduction in atrial and ventricular contractility. In isolated rat hearts, (-)-carvone caused a decrease in heart rates and increase in PR intervals, typical of calcium channel blockers. In addition, a significant reduction in the severity of arrhythmias such as ventricular fibrillation in hearts submitted to (-)-carvone perfusion was observed. (-)-Carvone is, therefore, a highly promising natural substance in respect of the development of new antiarrhythmic drugs.
